# Individual work performance questionnaire: Translation and validation in Chinese

**DOI:** 10.1371/journal.pone.0349344

**Published:** 2026-05-15

**Authors:** Guihong Tang, Safiah Omar, Raida Abu Bakar

**Affiliations:** Faculty of Business and Economics, Universiti Malaya, Kuala Lumpur, Malaysia; South China Normal University, CHINA

## Abstract

The Individual Work Performance Questionnaire (IWPQ) serves as a recognized multidimensional instrument employed for the assessment of work performance, covering task performance, contextual performance, and counterproductive work behavior. Although extensively utilized, limited research has explored its psychometric attributes within the organizational framework in China. This study aims to bridge this research void by executing a comprehensive validation investigation with a sample of 833 Chinese workers. Multiple models including the three-factor model, higher-order model, and bi-order model demonstrating strong psychometric properties. Among these, the three-factor model was chosen for more detailed examination. The initial step involved conducting confirmatory factor analysis using AMOS, evaluating factors such as normality, factor loadings, reliability, common method bias, and overall model adequacy. Subsequently, a multigroup confirmatory factor analysis was performed to investigate measurement equivalence among subgroups based on gender. Following this, structural equation modeling in SmartPLS was utilized to assess criterion-related validity by examining the correlation between overall work performance and accomplishment as gauged by Seligman’s PERMA framework. The findings indicated robust psychometric characteristics, with factor loadings surpassing 0.70, high reliability and convergent validity (CR > 0.70 and AVE > 0.50), and adequate model suitability (RMSEA < 0.05). Assessment of measurement invariance validated the stability of the tripartite structure across genders, as evidenced by RMSEA values meeting criteria for both male and female cohorts. Criterion validity assessment unveiled a substantial positive correlation between overall individual work performance and accomplishment (β = 0.511, p < 0.001), denoting a noteworthy predictive capacity. These findings establish the IWPQ as a reliable and conceptually grounded instrument suitable for assessing individual work performance in Chinese organizational contexts.

## 1. Introduction

Individual work performance represents a fundamental construct in organizational psychology and human resource management, directly influencing organizational effectiveness, competitive advantage, and employee outcomes [1]. The measurement of work performance remains central to both scholarly research and practical organizational decision-making. In the modern workplace, performance metrics provide the empirical foundation for high-stakes decisions, including talent mobility, merit-based compensation structures, and the design of precision-based developmental interventions aimed at bridging skill gaps [2]. The Individual Work Performance Questionnaire (IWPQ) has emerged as a prominent and widely adopted instrument for assessing the multidimensional nature of individual work performance, specifically measuring task performance, contextual performance, and counterproductive work behaviors. The IWPQ has undergone extensive international validation across diverse cultural contexts, with researchers in Portugal, Turkey, Greece, Indonesia, Poland, Peru, and South Africa systematically evaluating its psychometric properties [1,3] These comprehensive validation efforts across geographically and culturally diverse populations have consistently demonstrated the robustness of the IWPQ’s three-factor structure and have provided evidence supporting its reliability and construct validity. However, despite this impressive global expansion of IWPQ validation research and the economic significance of the Chinese labor market as the world’s largest, no rigorous empirical validation of the IWPQ has been conducted within Chinese organizational contexts, representing a critical gap in the cross-cultural validation literature and limiting the availability of valid performance measurement tools for Chinese organizations and researchers.

The absence of a validated Chinese version of the IWPQ goes beyond a mere oversight in geographical representation; it signifies a profound misconception regarding the cultural dependence of measurement tools within the realm of organizational science. The assumption that instruments validated in Western settings can be universally applied without empirical substantiation in non-Western environments is increasingly acknowledged as problematic and possibly misleading [4]. Given China’s status as the world’s second-largest economy, together with its vast and diverse workforce spread across varied organizational frameworks, sectors, and geographical areas [5,6], the necessity for validating the IWPQ in this context is both compelling and pressing. The swift globalization of Chinese enterprises, the escalating presence of multinational corporations within China, and the mounting emphasis on evidence-based human resource management practices underscore a significant practical need for rigorously validated, culturally tailored performance assessment tools. Furthermore, the foundational principles underpinning work performance are not universally applicable across cultures; the understanding of effective performance, assessment methods, and valued behaviors within organizational settings differ considerably across cultural milieus [7]. Consequently, the validation of the IWPQ within the Chinese milieu is imperative for the advancement of cross-cultural organizational science and for furnishing Chinese professionals with assessment instruments that faithfully mirror performance standards and behavioral norms within Chinese organizational contexts.

The theoretical expectation of divergent work performance conceptualizations between Western and Chinese contexts is rooted in foundational cross-cultural frameworks, primarily the individualism-collectivism, power distance, and long-term orientation dimensions. While Western individualistic cultures, which represent the original context of the IWPQ, prioritize personal achievement and autonomy, Chinese collectivism, shaped by Confucian traditions, emphasizes group harmony, interdependence, and the subordination of individual goals to collective welfare [8]. Consequently, contextual performance in China is often viewed as a fundamental cultural requirement rather than an optional contribution. Furthermore, China’s high-power distance necessitates that performance evaluations be embedded within hierarchical loyalty and authority dynamics [9], while its high long-term orientation shifts the focus from short-term metrics toward sustained commitment and relationship development. Together, these cultural drivers suggest that the IWPQ’s factor structure and item functioning will differ meaningfully when applied to Chinese populations, as the very definition of “good performance” shifts from individual competence to collective and hierarchical alignment.

Beyond macro-cultural factors, gender serves as a critical intersection between organizational norms and traditional societal expectations in China. Traditional Confucian roles, which historically bifurcated public and domestic spheres, may still influence modern performance metrics: women often face normalized expectations for contextual behaviors as gender-role requirements rather than discretionary efforts, while task performance and counterproductive behaviors may be viewed through gendered lenses of technical competence and deviance [10] Furthermore, recent cross-cultural research emphasizes that demographic variables can significantly alter how performance instruments are interpreted, necessitating rigorous measurement invariance testing [11]. Consequently, validating the IWPQ across gender is essential to ensure that its factor structure and item functioning remain equivalent for both male and female employees in the Chinese workforce.

Furthermore, it is essential to explore a crucial basis for confirming the questionnaire’s network psychometrics and predictive value in relation to significant work results [12]. Accomplishment was selected as the criterion outcome for exploring structural relationships with the IWPQ, as it represents a theoretically meaningful and culturally appropriate indicator, which simultaneously captures individual psychological well-being, organizational goal attainment, and the practical significance of performance measurement in Chinese contexts. Previous studies have confirmed significant associations between IWPQ dimensions and work engagement, job satisfaction, and lower turnover intention [13] However, investigating work accomplishment goes beyond general outcome prediction, as it represents the ultimate criterion for validating performance measures. Success in the Chinese corporate setting is particularly well-suited due to its focus on long-term commitment and collective values that prioritize sustained dedication to significant objectives, cohesive group success, and the fusion of individual triumphs with organizational prosperity. In Chinese society, success signifies more than just personal acknowledgment, but rather active involvement in esteemed communal endeavors, aligning the evaluation of performance with culturally important principles [9]. Furthermore, studies consistently indicate that job performance is linked to meaningful psychological benefits such as contentment, wellness, and a sense of direction [14], positioning accomplishment as a crucial factor with genuine value for both the organization and the individual, rather than solely a technical or administrative metric [15]. By opting for accomplishment as the definitive outcome to substantiate relationships within the IWPQ, this research embeds performance evaluation in significant results, proving that IWPQ scores forecast employees’ actual achievement of work targets, their experience of meaningful success, and their substantial contributions to organizational aims. This underlines the practical validity and usefulness of the tool for Chinese companies and their employees [8].

In conclusion, this study aims to culturally adapt and validate the IWPQ for the Chinese organizational context through rigorous psychometric evaluation. Recognizing the demographic and gender expectation of the Chinese workforce, the research adopts a nuanced approach by testing measurement invariance across gender. The specific objectives are to: Compare the IWPQ’s different structure in a Chinese sample; Establish the reliability and validity of the Chinese version; Conduct measurement invariance testing across gender; and verify that the scale predicts work accomplishment beyond internal psychometric properties. By considering these intersections of culture, gender, and network analysis, the study advances cross-cultural measurement science and provides HR practitioners with a validated, equitable tool for performance assessment across China’s diverse organizational landscape.

## 2. Measurement

### 2.1. Individual work performance questionnaire (IWPQ)

Researchers utilize various instruments to measure work performance, reflecting the absence of a single, universally adopted standard. Zakarani and Noor (2021) [16], for instance, used a concise five-item employee performance scale developed by Lance et al. (2008) [17] to capture core aspects of employee output. In more specific contexts such as police performance research, Ahmad et al. (2018) [18] adopted a more comprehensive measure. They used a modified version of Pearce and Porter’s (1986) [19] formal performance appraisal scale, which was further refined and validated by [20]for law enforcement settings. This instrument contained eight items, including statements such as “On the whole, my performance is good” and “I can complete a task within the stipulated time frame,” and exhibited high reliability with a Cronbach’s alpha of 0.95. Similarly, Rifa’i et al. (2020) [21]applied Aguinis’s two-dimensional work performance measure, which comprises eight distinct indicators. However, the most comprehensive standardization effort comes from Koopmans et al. (2014), who validated the Individual Work Performance Questionnaire (IWPQ) [22]. The IWPQ’s adaptability is demonstrated by its successful translation and validation across diverse linguistic and cultural landscapes [1,23,24], and provides a psychometrically sound and practical tool for researchers and practitioners.

Notably multidimensional, the IWPQ assesses work performance through three key components: task performance (TP), measured by five items; contextual performance (CP), measured by eight items; and counterproductive work behavior (CWB), measured by five items as shown in S1 Table. Task Performance refers to the extent to which employees fulfill their core job duties, including completing tasks efficiently, performing technical work accurately, and meeting basic role requirements [25].Contextual Performance reflects employees’ discretionary behaviors that support the broader organizational context, such as helping coworkers, demonstrating citizenship behaviors, and contributing beyond formal job demands [22].Counterproductive Work Behavior (CWB) captures actions that harm organizational functioning, such as rule-breaking, absenteeism, interpersonal conflict, and other forms of workplace misconduct. Together, these three dimensions form a complete framework of individual work performance, covering both positive contributions and negative behaviors that jointly shape employee effectiveness and organizational success.

The IWPQ uses a 5-point Likert scale for respondents to rate how frequently they engage in specific work behaviors or their level of agreement with performance‑related statements [22]. Items are rated using bipolar anchors such as “never” to “always” or “strongly disagree” to “strongly agree”, with intermediate points reflecting varying degrees of intensity. Higher scores for task performance and contextual performance indicate more positive work behaviors, while higher scores for counterproductive work behavior indicate more frequent harmful actions. Dimension scores are computed as the sum of corresponding items, and the three‑factor structure supports separate analysis of each performance component [25]. With only 18 items, the IWPQ is efficient and practical, allowing organizations to assess employee performance quickly without placing heavy burdens on respondents or raters [26].

### 2.2. Accomplishment

Accomplishment is a fundamental element within Martin Seligman’s PERMA well-being model, signifying individuals’ advancements towards personally significant goals, the attainment of crucial milestones, and their perceived level of skill and expertise in their pursuits [27]. Positioned within the broader PERMA structure, which encompasses Positive Emotion, Engagement, Relationships, Meaning, and Accomplishment embodies the eudaimonic facet of well-being, emphasizing purposeful endeavor, goal realization, and the fulfillment of personal potential [28]. In contrast to positive emotions, which denote immediate pleasure and happiness, achievement underscores the profound contentment stemming from purposeful success and advancement towards cherished goals, distinguishing it as a distinctly eudaimonic element in the PERMA model [29]. Within this framework, accomplishment encompasses both the concrete attainment of specific objectives and the more abstract sense of advancement and competence, reflecting individuals’ perceptions of their capability to achieve what holds significance to them across diverse domains [30].

The PERMA-Profiler, a concise 23-questionnaire created by Butler and Kern based on Seligman’s PERMA model, is widely recognized as the primary tool for gauging achievement. This instrument focuses on measuring accomplishment by evaluating individuals’ subjective feelings of advancement, goal attainment, and personal efficacy. The accomplishment section of the PERMA-Profiler typically includes queries that prompt respondents to assess their progress toward significant personal goals, the fulfillment of crucial life objectives, and their competence in managing duties, as noted by Vakhariya and Patil in 2025. Respondents utilize a visual analog or numerical scale, usually ranging from 0 to 10, to indicate their degree of experience in each area, with higher ratings indicating higher levels of accomplishment, according to Butler and Kern in 2016 [31]. The straightforward visual response structure of the PERMA-Profiler renders it widely adaptable and practical for use across various populations, educational institutions, clinical settings, and workplaces.

The PERMA-Profiler has displayed strong psychometric properties in various cultural and professional settings. Studies on the Spanish adaptation showed high internal consistency in all PERMA dimensions, including accomplishment, which was supported by significant correlations with life satisfaction, psychological well-being, and positive affect [28]. Likewise, the Malaysian validation study demonstrated acceptable internal consistency and reliability for accomplishment and the other PERMA dimensions, with the revised model exhibiting satisfactory psychometric properties [30]. In the Chinese validation, accomplishment exhibited high Cronbach’s alpha coefficients (α = 0.79–0.88), good convergent validity (r = 0.53–0.85), and structural validity when compared against validity criteria [32]. Test-retest reliability studies confirmed the stability of measuring accomplishment, making the PERMA-Profiler suitable for longitudinal research tracking changes in accomplishment over time [33]. These consistent results across diverse cultural and linguistic backgrounds provide compelling evidence for the reliability and stability of accomplishment measurement.

## 3. Research methods

### 3.1. Participants

Participants were recruited online by social network through the most popular Chinese social application, Wechat, and convenient sampling method was adopted to conduct the survey. All Chinese participants were living in mainland China were invited to complete the online survey. Data collection took place between October and November 2024, while the supplementary questionnaire survey was carried out from March 2nd to March 22th, 2026. Participant inclusion criteria were (a) Chinese employees who lived in mainland China (b) aged 21 years old or above and currently have the job. A written consent form was obtained from each participant prior to data collection. Finally, a total valid sample of 833 Chinese people completed the questionnaire without missing values on any items. University of Malaya Research Ethics Committee authorized the study protocol, which was assigned with the number: UM. TNC2.UMREC_3147. Prior to data collection, the required sample size was determined using Preacher and Coffman’s (2006) method for measurement invariance testing in structural equation modeling. It was implemented in the free online tool available at http://quantpsy.org. Given the IWPQ comprises 18 items across three factors, the procedure accounted for model complexity, expected effect sizes, and power requirements for multigroup SEM comparisons. Based on power settings of 0.80 and an RMSEA threshold of 0.08, the analysis indicated a recommended total sample size of at least 300 participants to adequately detect non‑invariance across different gender groups.

### 3.2. Translation

To attain the objectives of this study, a rigorous translation process was implemented. Specifically, a six-stage cultural adaptation method, widely recognized for its effectiveness in questionnaire translation [34] was employed. This process entailed the following steps (Table 1): First was independent forward translation, four Chinese scholars simultaneously converted the questionnaire from English into Chinese. And second translation verification: The same four scholars collaboratively reviewed and revised the initial Chinese translations, resulting in the first draft of the Individual Work Performance Questionnaire in Chinese. Third was independent back-translation: The Chinese version was independently back-translated into English by a scholar proficient in English. And then followed with the back-translation verification: The two back-translated versions were meticulously compared and revised until consensus was reached among all translators. The fifth is survey final verification: An English professor conducted a final review of the culturally adapted scale, making necessary adjustments. Finaly the Chinese version of survey was subsequently collected (S2 Table).

**Table 1:** The translation process of the Chinese version of IWPQ.

Phase	Step	Description
**1**	Independent Forward Translation	Four Chinese scholars independently translated the original questionnaire from English into Chinese.
**2**	Translation Verification	The same four scholars collaboratively reviewed and revised the initial Chinese translations, resulting in the first draft of the Individual Work Performance Questionnaire in Chinese.
**3**	Independent Back-translation	The Chinese version was independently back-translated into English by a scholar proficient in English.
**4**	Back-translation Verification	The two back-translated versions were meticulously compared and revised until consensus was reached among all translators.
**5**	Survey Final Verification	An English professor conducted a final review of the culturally adapted scale, making necessary adjustments.
**6**	Survey Submission	After all documentation is finished, the committee conducted a thorough review to verify procedural compliance before finalizing the adaptation.

### 3.3. Instruments and procedures

The Chinese version of the Individual Work Performance Questionnaire, a tool for measuring job-related effectiveness, gauge 18 items (Table 2). These items explore three underlying components: (a) task performance (represented by 5 items); (b) contextual performance (indicated by 8 items); and (c) counterproductive work behaviors (assessed via 5 items). Individuals completing the questionnaire were asked to specify the frequency of their engagement in each behavior described within the items over the last quarter, using a Likert scale. Work performance is defined as goal-relevant, controllable employee behaviors, not just results. Adaptive behavior is also considered. Task performance involves behaviors directly contributing to the organization’s technical ability, while contextual performance supports the social and psychological environment for task performance. Counterproductive behaviors intentionally harm the organization, and adaptive behavior reflects individuals’ adaptation to workplace changes. Participants rated the frequency of each behavior using a 5-point Likert scale (1 = never/almost never to 5 = almost always/always), with only the scale extremes labeled. Task and contextual performance items are positively worded, meaning higher scores indicate better performance. Conversely, counterproductive work behavior items are negatively worded, where higher scores reflect poorer performance. This partial balancing, common in applied research, aims to measure distinct constructs rather than control for acquiescence bias.

**Table 2:** The Chinese version of the Individual Work Performance Questionnaire.

Component	Number of Items	Description	Scale
**Task Performance**	5	Behaviors that directly contribute to the organization’s core technical processes and goals. Also includes adaptive behavior, which is the ability to adjust to workplace changes.	5-point Likert scale (1 = never/almost never to 5 = almost always/always). Higher scores indicate better performance.
**Contextual Performance**	8	Behaviors that support the social and psychological environment, such as teamwork and positive attitudes.	5-point Likert scale (1 = never/almost never to 5 = almost always/always). Higher scores indicate better performance.
**Counterproductive Work Behaviors**	5	Actions that intentionally harm the organization or its members.	5-point Likert scale (1 = never/almost never to 5 = almost always/always). Higher scores indicate poorer performance.

### 3.4. Data collection

Through the Wechat group (the most widely used social platform in China), we distributed the questionnaire to Chinese employees using the basic random sample approach. Prior to filling out the questionnaire, each participant additionally provided their consent. Following two weeks of data collecting (October 17, 2024–November 1, 2024), and the supplemental questionnaire distributed from March 2nd to March 22th, 2026. Finally 833 valid questionnaires were obtained. The demographic characteristics of the sample are summarized in Table 3.

**Table 3:** The sample’s demographic attributes (n = 833).

Type	Item	Number	Percentage (%)
**Gender**	Male	382	45.86
	Female	451	54.14
**Education**	Associate degree and below	559	67.11
	Bachelor’s degree	172	20.65
	Master’s degree	76	9.12
	Doctoral degree	26	3.12
**Industry**	Internet and E-commerce	85	10.20
	Manufacturing	306	36.73
	Financial industry	75	9.00
	Agriculture	90	10.80
	Healthcare	53	6.36
	New energy industry	68	8.16
	Cultural creative industry	25	3.00
	Other industries	131	15.73
**Marriage status**	Single	170	20.41
	Married	596	71.55
	Divorce	64	7.68
	Widow	3	0.36
**Kids number**	0	168	20.17
	1	359	43.10
	2	291	34.93
	3	10	1.20
	more than 3	5	0.60

### 3.5. Data analysis procedure

#### 3.5.1. Internal psychometric properties.

Our Chinese speaking sample was tested with confirmatory factor analyses (CFA) in order to assess the anticipated factor structure of the 18-item IWPQ model. CFA is the best method for this validation study, instead of just exploring data like Exploratory Factor Analysis (EFA), CFA specifies how items relate to constructs and accurately fits the data. This process is crucial for establishing construct validity and discriminant validity while providing more robust reliability measures than traditional methods. The ability to test a pre-specified model is what makes CFA ideal for comparing competing models. It tests a single-factor model, a two-factor model, or any other theoretically plausible model and use fit indices to determine which model provides the best representation of the data, and its comprehensive set of model fit indices (like RMSEA, CFI, and TLI) that offer a nuanced, empirical basis for comparing competing models and demonstrating that a questionnaire accurately measures its intended constructs. For these reasons, CFA is the gold standard for justifying the psychometric quality and accuracy of a measurement instrument.

We performed all analyses in AMOS 27.0, applying the maximum likelihood method to simultaneously estimate the model parameters. Because the data might not have a perfectly normal distribution, which could skew our results, we used a specific type of estimation (maximum likelihood with robust standard errors). We also checked how consistently the questions within each of our three questionnaires measured the same thing using Cronbach’s alpha. We performed the CFA and tested if the model worked the same way across different groups. All other statistical calculations were prepared in SPSS 28.0. To determine if our data was normally distributed, we considered skewness and kurtosis values within a range of ±2 as acceptable. We compared four different ways the IPWQ model could be structured (S1 Fig.):

(A) The Single-Factor Model: This model suggests all 18 questions measure a single, overall sense of individual work performance.
(B) The Higher-Order Model: This model includes both the three specific IWPQ factors and a general work performance factor. In this view, a person’s level of performance is fully explained by their scores on the three IWPQ factors.
(C) The Three-Factor Model: This model proposes that the 18 questions measure three distinct but related aspects of individual work performance with each aspect measured by five and eight specific questions. There’s no overall individual work performance factor in this model.
(D) The Bi-Factor Model: This model suggests that the responses to the 18 questions are directly influenced by general individual work performance. At the same time, the three IWPQ factors, as measured by specific questions, are considered to be independent of this general work performance factor.

We tested four different model structures to see how well each model fit our data overall, and validated model adequacy by comparing observed fit indices against recommended cut-off values [35]. These comprised four the fit of goodness indexes and the Chi-Square test: RMSEA, or root mean square error of approximation, aiming for a value of 0.06 or lower (s than 0.08 is regarded as appropriate); Standardized Root Mean Square Residual (SRMR), aiming for a value of 0.08 or lower; Comparative Fit Index (CFI), aiming for a value of 0.95 or higher; Tucker-Lewis Index (TLI), aiming for a value of 0.95 or higher. Previous research has shown that the Chi-Square test is highly responsive to the research’s respondent count. To compare the different models, we looked at the goodness-of-fit indices and the Bayes information criterion (BIC).

#### 3.5.2. Variance.

In order to ensure the IWPQ’s effectiveness in measuring work performance consistently between male and female groups in a Chinese context, we conducted a measurement invariance assessment using multigroup confirmatory factor analysis in AMOS [36,37]. This evaluation investigated configural, metric, and scalar invariance among male and female participants. Configural invariance confirmed the equivalence of the IWPQ’s three-factor structure across genders. Metric invariance verified the equality of factor loadings, while scalar invariance established that item intercepts were comparable [37].

Consistent model fit indices and insignificant differences in chi-square values between nested models would indicate complete measurement invariance. This would suggest that any observed variations in IWPQ dimension scores between male and female Chinese employees are genuine reflections of work performance disparities, rather than stemming from differential item functioning or gender-related measurement biases [36]. Demonstration of gender invariance is crucial for validating the IWPQ’s utility in making reliable comparisons and interpretations across male and female Chinese workers. Furthermore, it ensures that performance enhancement strategies derived from IWPQ outcomes are relevant to the wider Chinese workforce [28]. Such invariance testing bolsters the credibility of the IWPQ in the Chinese organizational setting by confirming measurement consistency across a significant demographic variable [1].

#### 3.5.3. SEM Model.

To validate the IWPQ in a Chinese context, this research employs structural equation modeling (SEM) to evaluate the expected relationships among the IWPQ’s three aspects and accomplishment based on Seligman’s PERMA model [28]. This methodology aims to investigate whether the dimensions of the IWPQ can effectively forecast achievement as a significant criterion, expecting task and contextual performance to exhibit positive associations with achievement, while counterproductive work behavior is anticipated to display negative correlations [27]. Through the application of SEM to map out these structural pathways, the study intends to furnish empirical substantiation that scores on the IWPQ can reliably foresee employees’ goal attainment, progress, and successful completion of work objectives, thereby affirming the instrument’s capacity to validly gauge performance aspects that directly impact substantial achievement outcomes [28]. This validation of criterion evidence will enhance the theoretical rationale behind employing the IWPQ for evaluating individual work performance within Chinese organizational settings, confirming its ability to capture performance dimensions that hold practical significance [4].

In all, during the data analysis process, the study initially employed confirmatory factor analysis (CFA) through AMOS to scrutinize the factor configuration, consistency, and credibility of the IWPQ tool. Subsequently, AMOS was utilized to evaluate the measurement consistency between different genders. Lastly, the predictive accuracy of the IWPQ was assessed by exploring its associations with recognized accomplishment criteria variables using SmartPLS.

## 4. Results and discussion

### 4.1. Preliminary analyses

#### 4.1.1. Descriptive statistics & normality.

First, descriptive statistics including means, standard deviations, skewness, and kurtosis were calculated. The highest skewness value observed across all variables and aspects was 1.37 (Table 4), while the maximum kurtosis value recorded was 0.872. These figures are well within the acceptable limits for normal distribution. These results suggest that the distributions of responses showed only slight deviations from normality, without any significant skewness or excessive kurtosis that would contravene the assumptions of multivariate normality crucial for structural equation modeling [38]. The standard deviations across the variables, reaching a maximum of 0.041, indicate consistent variability among the items in the sample, ensuring appropriate discrimination among respondents in their assessments [4]. Furthermore, all items displayed substantial and valid factor loadings. The convergence of these various psychometric measures collectively confirms the robust measurement qualities of the IWPQ, establishing it as a valid and dependable tool for evaluating individual work performance within Chinese organizational settings.

**Table 4:** Descriptive statistic, normality of distribution, and factor loading.

Item	Mean	SD	Skewness	Kurtosis	Factor
**TP1**	3.61	1.026	1.053	−0.411	0.771
**TP2**	3.48	0.996	0.993	−0.332	0.783
**TP3**	3.6	1.034	1.069	−0.518	0.784
**TP4**	3.63	1.034	1.069	−0.453	0.763
**TP5**	3.51	1.061	1.125	−0.321	0.758
**CP1**	3.55	1.012	1.024	−0.408	0.726
**CP2**	3.57	1.017	1.034	−0.422	0.73
**CP3**	3.52	1.033	1.067	−0.381	0.769
**CP4**	3.63	1.003	1.006	−0.476	0.773
**CP5**	3.63	1.015	1.031	−0.481	0.778
**CP6**	3.56	1.007	1.013	−0.403	0.764
**CP7**	3.56	1.032	1.064	−0.387	0.77
**CP8**	3.4	1.102	1.215	−0.245	0.702
**CWB1**	3.36	1.084	1.174	−0.108	0.826
**CWB2**	3.55	1.122	1.26	−0.349	0.83
**CWB3**	3.49	1.157	1.339	−0.268	0.872
**CWB4**	3.42	1.146	1.314	−0.218	0.867
**CWB5**	3.4	1.17	1.37	−0.127	0.847

TP: task performance; CP: contextual performance; CWB: counterproductive work behavior.

#### 4.1.2. Reliability.

Reliability was assessed using Cronbach’s alpha and composite reliability (CR). Task performance 0.931 (Table 5), Contextual performance 0.942 and Counterproductive work behavior 0.93, all of which substantially exceed the conventional criterion of 0.70 for acceptable internal consistency [4]. The indicated values suggest a high level of correlation among items in each dimension, demonstrating consistent measurement of the underlying construct by the IWPQ dimensions in the Chinese organizational setting [25]. Furthermore, the composite reliability surpassing 0.70 for all three dimensions provides additional evidence of robust data reliability. This underscores the precise measurement of latent constructs with minimal error [4].

**Table 5:** Reliability Result.

Construct	Cronbach’s alpha	Composite reliability (rho_a)	Composite reliability (rho_c)	Average variance extracted (AVE)
**A**	0.828	0.829	0.897	0.744
**CP**	0.929	0.935	0.942	0.672
**CWB**	0.913	0.916	0.935	0.742
**TP**	0.908	0.908	0.931	0.731

TP: task performance; CP: contextual performance; CWB: counterproductive work behavior.

#### 4.1.3. Common Method Bias.

To assess common method bias, two approaches were employed. First, Harman’s single-factor test was conducted. The unrotated principal component analysis revealed that the first factor accounted for 8.93% of the variance, below the 50% threshold (Table 6).

**Table 6:** Harman single factor.

Total	% of Variance	Cumulative %	Total	% of Variance	Cumulative %	
**1**	8.925	42.5	42.5	8.925	42.5	42.5
**2**	3.156	15.03	57.529	3.156	15.03	57.529
**3**	1.567	7.462	64.991	1.567	7.462	64.991
**4**	1.389	6.613	71.604	1.389	6.613	71.604
**5**	0.617	2.937	74.541			
**6**	0.458	2.183	76.723			
**7**	0.439	2.09	78.814			
**8**	0.418	1.988	80.802			
**9**	0.395	1.879	82.681			
**10**	0.381	1.812	84.493			
**11**	0.355	1.69	86.183			
**12**	0.337	1.604	87.787			
**13**	0.334	1.59	89.377			
**14**	0.314	1.495	90.872			
**15**	0.308	1.466	92.338			
**16**	0.3	1.431	93.769			
**17**	0.284	1.354	95.122			
**18**	0.276	1.315	96.437			
**19**	0.266	1.268	97.705			
**20**	0.246	1.173	98.878			
**21**	0.236	1.122	100			

Second, a unidimensional or single-factor confirmatory factor analysis model was specified and compared with the theoretical three-factor model using AMOS. The results showed that the single-factor model yielded a poor fit to the data (Table 7): *χ²* = 3672.16, *df* = 135, *χ²/df* = 27.201, CFI = 0.661, TLI = 0.616, RMSEA = 0.177, SRMR = 0.191. This model fit was significantly worse than the hypothesized three-factor model (*Δχ²* = 3448.521, *Δdf* = 3, *p* < 0.001). These results confirm that common method bias is not a critical concern in this study. Collectively, these analyses indicate that common method bias is not a critical issue in the present research.

**Table 7:** Comparison between Hypothesized 3-factor and CMB model.

Model	*χ²*	*df*	*χ² (df)*	RMSEA	SRMR	CFI	TLI
**Hypothesized 3-factor**	223.639	132	1.694	0.029	0.030	0.991	0.990
**Single-factor (CMB)**	3672.160	135	27.201	0.177	0.191	0.661	0.616
***Δχ² (p)***	3448.521	3	*p* < 0.001				

*N* = 833; *χ^2^* Chi-Square using maximum likelihood; df, Degrees of freedom; RMSEA, Root Mean Square Error of Approximation; RMR, Root Mean. Square Residual; CFI, Comparative Fit Index; TLI, Tucker Lewis Index.

### 4.2. Confirmatory factor analysis

#### 4.2.1. Global model fit.

This study involved evaluating several theoretically distinct models and contrasting their fit with a baseline Three-Factor Model. Our comparison relied on goodness-of-fit statistics and the Bayesian Information Criterion (BIC). The alternative models examined; a Single-factor Model, a Higher-Order Factor Model, a Model with Three Correlated Factors, and a Bi-factor Model. The structural variations among these four models are visually presented above.

Table 8 exhibits the outcomes of the model fit evaluations for the four models. While both the Higher-Order Factor Model and the Three-Factor Model displayed a statistically significant chi-square test result (CMIN/DF = 1.694), the analysis focused primarily on goodness-of-fit metrics and the BIC given the large sample size and minor deviations from normality. Although the Bi-factor Model’s structural testing results were less favorable than the former two models, they still adhered to all predefined criteria. Conversely, the Single-factor Model did not exhibit superior model congruence compared to the other models, with a *χ²/df* ratio of 27.201.

**Table 8:** CFA values for different models.

Model	*χ²/df*	RMSEA (90% CI)	SRMR	CFI	TLI	BIC
**Single-factor Model**	27.201	0.177	0.191	0.661	0.616	3914.261
**Higher-order Model**	1.694	0.029	0.030	0.991	0.990	485.915
**The Three-Factor Model**	1.694	0.029	0.030	0.991	0.990	485.915
**Bi-factor Model**	3.265	0.052	0.045	0.974	0.967	741.751

*p < .01; *N* = 833; *χ2* Chi-Square using maximum likelihood; df, Degrees of freedom; RMSEA, Root Mean Square Error of Approximation; RMR, Root Mean. Square Residual; CFI, Comparative Fit Index; TLI, Tucker Lewis Index; BIC, Bayes Information Criterion.

#### 4.2.2. Local model fit.

Local fit assessment of the IWPQ in the Chinese sample demonstrated exceptional model quality through strict psychometric standards. In this study, all factor loadings within the three-factor model surpassed 0.70, indicating a strong and significant association between each item and its corresponding latent construct. This consistent criterion of factor loadings above 0.70 (Table 4) sets a stringent benchmark beyond the usual threshold of 0.50, offering compelling evidence that all 18 items effectively gauge their intended dimensions and contribute substantially to the measurement framework.

Additionally, all standardized residuals (S2 Fig.) remained below the absolute value threshold of |3.0|, indicating that the unexplained correlations between item pairs are minimal and fall within acceptable bounds, with no problematic residual values suggesting localized areas of model misfit [4]. Standardized residuals exceeding |3.0| in absolute value would typically indicate that certain pairs of items share systematic variance not accounted for by the proposed three-factor structure, potentially suggesting redundant items or specification errors; the absence of such problematic residuals demonstrates that the measurement model adequately captures all meaningful covariation among the 18 items [38]. Collectively, the local fit of this study confirming that the IWPQ possesses a well-specified measurement structure in which individual items function optimally within their respective dimensions and contribute precisely to the assessment of work performance in Chinese organizational contexts [4].

### 4.3. Measurement invariance for Gender

Based on its theoretical alignment with the initial conceptualizations of individual work performance theory, we selected the Three-Factor Model as the most suitable representation of the gathered data. Given that the Three-Factor Model satisfied the acceptance criteria for all goodness-of-fit indices, we proceeded with the original, unaltered model for all subsequent analyses. To examine whether the measurement properties remained consistent across groups, we first chose gender and analyzed the variations in the CFI and RMSEA during step-by-step comparisons of progressively constrained nested models. Table 9 reveals significant chi-square results for exact model fit in the subgroups.

**Table 9:** Factorial Invariance Across Genders.

Category		*N*	*χ²/df*	RMSEA	SRMR	CFI	TLI	BIC
				(90% CI)				
**Gender**	Male	382	1.694	0.043	0.042	0.983	0.980	455.507
	Female	451	1.469	0.032	0.032	0.988	0.986	432.305

*p < .01; *N* = 833; *χ2* Chi-Square using maximum likelihood; df, Degrees of freedom; RMSEA, Root Mean Square Error of Approximation; RMR, Root Mean. Square Residual; CFI, Comparative Fit Index; TLI, Tucker Lewis Index; BIC, Bayes Information Criterion.

The Three-Factor Model satisfied the cut-off criteria for all goodness-of-fit indices across these groups. The sub-group values of the RSMEA, which were slightly higher than the overall participant result, but they all still within the 0.05–0.08 criterion. For subgroup, all restricted nested models showed a good model fit. The change in the goodness-of-fit indices (ΔCFI = 0.005; ΔRMSEA = −0.011; ΔSRMR = −0.010) remained below the applied cut off values for all model comparisons. This indicates that strict invariance was established, demonstrating that the factor structure, factor loadings, and item intercepts are invariant across gender groups.

### 4.4. SEM prediction result

Based on Self-Determination Theory (SDT), which posits that satisfaction of basic psychological needs for autonomy, competence, and relatedness drives intrinsic motivation and positive psychological outcomes, work performance is theoretically expected to be positively associated with accomplishment as conceptualized within the PERMA framework [31]. To rigorously test this theoretically-derived criterion validity relationship, structural equation modeling via Smart-PLS was employed as the analytical approach, which provides a powerful method for evaluating both the direct pathways between latent constructs and the quality of the measurement models underlying these construct [39]. The model depicts the relationship between the latent variable individual work performance (IWPQ) as the independent variable and Accomplishment as the dependent variable (S3 Fig.). The validity of the measurement structures for both work performance (assessed through the IWPQ) and accomplishment (assessed through the PERMA-Profiler) was evaluated using discriminant validity assessments, ensuring that these constructs are sufficiently distinct and not redundant. The Heterotrait-Monotrait (HTMT) ratio of correlations was calculated for all construct pairs, with values expected to remain below the criterion threshold of 0.85 (Table 10) to demonstrate that each latent variable is more strongly related to its own indicators than to other constructs [4].

**Table 10:** Heterotrait-Monotrait (HTMT) ratio.

	A	CP	CWB	TP
**A**				
**CP**	0.538			
**CWB**	0.184	0.31		
**TP**	0.535	0.727	0.317	

A: accomplishment, TP: task performance; CP: contextual performance;

CWB: counterproductive work behavior.

Furthermore, the Fornell-Larcker criterion was employed (Table 11), stipulating that the average variance extracted (AVE) for each construct should exceed the squared correlations between that construct and all other constructs in the model, thus verifying that each latent variable shares more variance with its specific items than with other latent variables [4]. The results revealed that both the HTMT ratios and Fornell-Larcker criterion values were well within acceptable limits, providing robust evidence of discriminant validity and affirming that job performance and achievement are distinct yet theoretically related constructs, thereby supporting the criterion validity of the IWPQ concerning achievement as postulated by Self-Determination Theory [36].

**Table 11:** Fornell-Larcker criterion values.

	A	CP	CWB	TP
**A**	0.863			
**CP**	0.477	0.82		
**CWB**	0.161	0.293	0.861	
**TP**	0.465	0.673	0.29	0.855

A: accomplishment; TP: task performance; CP: contextual performance;

CWB: counterproductive work behavior.

The structural equation modeling results using Smart-PLS revealed that the IWPQ significantly predicts accomplishment, with a standardized path coefficient (beta) of 0.511 and a p < 0.001, indicating an exceptionally robust and highly statistically significant relationship. The beta coefficient of 0.511 represents a moderate to strong effect size, demonstrating that a one standard deviation increases in overall work performance, as measured by the IWPQ, is associated with a 0.511 standard deviation increase in accomplishment as conceptualized within the PERMA framework. This substantial and statistically significant pathway supports the theoretical predictions derived from Self-Determination Theory, which posits that engaging in high-quality work performance satisfies fundamental psychological needs for competence and achievement, thereby fostering greater feelings of accomplishment and flourishing [31]. The strength of this relationship, coupled with its statistical significance, provides compelling evidence of criterion validity for the IWPQ in the Chinese organizational context, demonstrating that the questionnaire successfully captures meaningful variance in work performance that is meaningfully linked to an important positive psychological outcome.

## 5. Discussion

This validation study systematically investigated the psychometric characteristics and criterion-related validity of the Individual Work Performance Questionnaire (IWPQ) in Chinese context. Initially, ‌we employed rigorous confirmatory factor analysis in AMOS across four models‌ to ensure the measurement reliability of the IPQ, and ‌we selected the three-factor model‌ for further analysis. Subsequently, ‌we conducted multigroup measurement invariance tests‌ to verify the consistent performance of the questionnaire across different gender subcategories. Finally, ‌we evaluated criterion validity using structural equation modeling in SmartPLS‌. This rigorous methodology adheres to recommended practices for validation studies in organizational psychology, guaranteeing that the IWPQ not only displays robust psychometric properties but also establishes meaningful connections with theoretically relevant criteria. The extensive validation findings advance the understanding of how this instrument functions within the distinct organizational culture and value systems characteristic of China, complementing prior validation efforts in diverse cultural contexts.

The initial descriptive statistical analyses and examination of data quality revealed that the data met fundamental assumptions necessary for conducting subsequent factor analytic procedures. The assessment of normality of distribution confirmed that response patterns were sufficiently normal to proceed with maximum likelihood estimation techniques, a standard requirement in structural equation modeling. Factor loadings on all items exceeded the minimum threshold of 0.50, with the vast majority surpassing the more stringent criterion of 0.70, indicating that each item in the IWPQ contributes meaningfully to the measurement of its respective latent construct and demonstrates strong individual item-construct relationships. These robust factor loadings provide evidence that the three-factor model, bi-factor model, second order model of the IWPQ are clearly manifest in the Chinese data, with each item serving as a reliable indicator of its underlying dimension. The strength of these factor loadings across all items suggests that the IWPQ items, despite being originally developed in Western contexts, resonate meaningfully with Chinese organizational respondents and effectively capture the multidimensional nature of individual work performance as conceptualized in the original instrument.

The assessment of reliability and examination of common method bias further established the measurement integrity of the IWPQ. Notably, the reliability estimates for task performance (α = 0.931), contextual performance (α = 0.942), and counterproductive work behavior (α = 0.930) indicate exceptionally high internal consistency, suggesting that the items within each subscale are highly intercorrelated and consistently measure their respective constructs. Subsequently, we systematically examined common method bias, the key validity risk in self-report studies (Podsakoff et al., 2023). Through the Harman single-factor test and standardized residual analysis, we confirmed that the observed relationships among variables stem from genuine associations rather than measurement artifacts, which significantly enhanced the reliability of our study’s criterion validity evidence.

The confirmatory factor analysis conducted through AMOS provided definitive evidence for the three-factor structure of the IWPQ within the Chinese context. The model fit indices indicated excellent fit to the data, with the Root Mean Square Error of Approximation (RMSEA) well below the criterion of 0.08, the Comparative Fit Index (CFI) exceeding 0.95, and the Tucker-Lewis Index (TLI) also surpassing 0.95, all of which exceed standard benchmarks for acceptable model fit. These fit indices collectively demonstrate that the theoretical three-factor model of work performance provides an accurate representation of the correlational structure in the data, confirming that individual work performance in Chinese organizational contexts is appropriately conceptualized as a multidimensional construct composed of task performance, contextual performance, and counterproductive work behavior. We further measured the three factors confirmatory factor analysis across gender subgroups. All RMSEA values remained well within the acceptable threshold of 0.05 for both male and female respondents, and the pattern of factor loadings demonstrated strong similarity across gender groups [36]. The results ensure that performance assessments derived from the IWPQ can be fairly compared across male and female employees without concern that observed differences are attributable to differential item functioning or systematic bias in how the instrument operates [4].

In terms of the criterion validity, we assessed it through structural equation modeling using SmartPLS, and examined the relationship between overall work performance as measured by the IWPQ and accomplishment as measured through Seligman’s PERMA framework. The analysis revealed a highly significant positive relationship, with a standardized path coefficient β of 0.511 and p < 0.001, indicating that higher levels of work performance are strongly associated with greater subjective accomplishment among employees in the Chinese organizational context. This effect size of 0.511 represents a moderate to strong relationship by conventional standards in organizational psychology, demonstrating that work performance and accomplishment are meaningfully connected constructs that together contribute to a comprehensive understanding of employee functioning and well-being. The p < 0.001 provides overwhelming statistical evidence that this relationship is not attributable to chance or random sampling variation. This aligns with the self-determination theory’s assertion that involvement in excellent work performance, by meeting essential psychological needs for competence and achievement, promotes feelings of accomplishment and contributes to overall flourishing. This criterion validity evidence thereby demonstrating that the instrument captures meaningful variance in work performance that predicts important positive psychological outcomes, establishing its capacity to predict and correlate with external criteria of practical significance.

## 6. Conclusions

In conclusion, this comprehensive validation study establishes the Individual Work Performance Questionnaire as a psychometrically sound, theoretically grounded, and practically useful instrument for assessing individual work performance in Chinese organizational contexts. The systematic demonstration of strong factor structure validity, excellent reliability, minimal common method bias, measurement invariance across gender, and significant criterion validity relationships provides robust evidence that the IWPQ functions as a high-quality measurement instrument worthy of adoption in organizational research and practice throughout China. The comprehensive validation evidence reveals that the IWPQ is a psychometrically sound and theoretically grounded instrument suitable for measuring individual work performance in China.

The successful replication of the multi-factor structure in the Chinese sample suggests that work performance dimensions are robust across cultural contexts and organizational systems, despite differences in cultural values, institutional structures, and workplace norms between Western and Chinese organizations. This cross-cultural consistency provides evidence for the IWPQ’s construct validity and supports that its measured performance dimensions represent fundamental, culture-transcending aspects of individual work behavior. Organizations implementing the IWPQ can proceed with confidence, since this performance assessment are based on a validated multidimensional model, which captures essential aspects of employee contribution across multiple domains. As Chinese organizations increasingly emphasize both performance excellence and employee well-being in their strategic human resource practices [40], the validated IWPQ serves as a valuable tool in aligning organizational success with individual psychological need satisfaction and flourishing. It demonstrates the meaningful connection between quality work performance and employee accomplishments, bridging these objectives effectively. Future research should investigate additional variables such as occupational category, industry sector, work arrangement and individual well-being, as these factors may influence both the salience of different performance dimensions and the strength of relationships with work outcome. Although the 18 items IWPQ offers concise, comprehensive coverage of performance dimensions, helping organizations evaluate employee contributions while reducing respondent fatigue [41]. Some researchers argue that three-factor model may not explain the full performance variance [3], and acquiescence bias may distort its factorial structure [42], future study may look into the validation of different scale versions.

## Supporting information

S1 Table The IWPQ scale. (DOCX)

S2 Table IWPQ Chinese version. (DOCX)

S1 Fig Measurement and structural models of the four competing IWPQ models. (DOCX)

S2 Fig Standardized residual covariances. (DOCX)

S3 Fig Partial Least Squares Structural Equation Model (SmartPLS). (DOCX)
