# Supplementary material for: Individual work performance questionnaire: Translation and validation in Chinese
Source: PLoS One. 2026 May 15;21(5):e0349344. doi: 10.1371/journal.pone.0349344 (PMC13178909; doi:10.1371/journal.pone.0349344)
Supplement: S1 Table — (DOCX) [file pone.0349344.s001.docx]

**S1 Table. The IWPQ scale.**

| **Dimension** | **Item** | **Description** |
| --- | --- | --- |
| **Task performance (TP)** | TP1 | I managed to plan my work so that it was done on time. |
|  | TP2 | My planning was optimal. |
|  | TP3 | I kept in mind the results that I had to achieve in mywork. |
|  | TP4 | I was able to separate main issues from side issues at work. |
|  | TP5 | I was able to perform my work with minimal time and effort. |
| **Contextual performance**  **(CP)** | CP1 | I took on extra responsibilities. |
|  | CP2 | I started new work myself when my old ones were finished. |
|  | CP3 | I took on challenging work tasks, when available. |
|  | CP4 | I worked at keeping my job knowledge up to date. |
|  | CP5 | I worked at keeping my job skills up to date. |
|  | CP6 | I came up with creative solutions to new problems. |
|  | CP7 | I kept looking for new challenges in my job. |
|  | CP8 | I actively participated in work meetings. |
| **Counterproductive behavior**  **(CWB)** | CWB1 | I complained about unimportant matters at work. |
|  | CWB2 | I made problems greater than they were at work. |
|  | CWB3 | I focused on the negative aspects of a work situation, instead of on the positive aspects. |
|  | CWB4 | I spoke with colleagues about the negative aspects of my work. |
|  | CWB5 | I spoke with people from outside the organization about the negative aspects of my work. |
