# Supplementary material for: Individual work performance questionnaire: Translation and validation in Chinese
Source: PLoS One. 2026 May 15;21(5):e0349344. doi: 10.1371/journal.pone.0349344 (PMC13178909; doi:10.1371/journal.pone.0349344)
Supplement: S2 Table — (DOCX) [file pone.0349344.s002.docx]

**S2 Table. IWPQ Chinese version.**

| **维度** | **序号** | **问题** |
| --- | --- | --- |
| **任务绩效** | TP1 | 我会提前计划工作，以便按时完成。 |
|  | TP2 | 我的计划是最佳的。 |
|  | TP3 | 我牢记我在工作中必须达到的结果。 |
|  | TP4 | 我能把工作中的主要问题和次要问题区分开来。 |
|  | TP5 | 我能用最少的时间和精力把工作做好。 |
| **关联绩效** | CP1 | 我在工作中承担了额外的责任。 |
|  | CP2 | 当一件任务完成后，我会自觉开始新的任务。 |
|  | CP3 | 只要有机会，我就承担具有挑战性的工作任务。 |
|  | CP4 | 我努力使自己的工作知识与时俱进。 |
|  | CP5 | 我努力使自己的工作技能与时俱进。 |
|  | CP6 | 我能对新问题提出创造性的解决方案。 |
|  | CP7 | 我一直在工作中寻找新的挑战。 |
|  | CP8 | 我积极参加工作会议。 |
| **反效行为** | CWB1 | 我抱怨工作中不重要的事情。 |
|  | CWB2 | 我制造了超出工作本身的麻烦。 |
|  | CWB3 | 我更关注工作环境的消极方面，而不是积极方面。 |
|  | CWB4 | 我会和同事谈论我工作中的消极方面。 |
|  | CWB5 | 我会和公司外的人谈论了我工作中的消极方面。 |
