# Supplementary material for: Individual work performance questionnaire: Translation and validation in Chinese
Source: PLoS One. 2026 May 15;21(5):e0349344. doi: 10.1371/journal.pone.0349344 (PMC13178909; doi:10.1371/journal.pone.0349344)

**S1 Fig. Measurement and structural models of the four competing IWPQ models.**

**S1 Fig. A.**   **S1 Fig. B.**


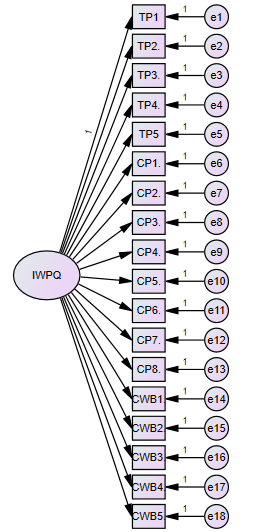

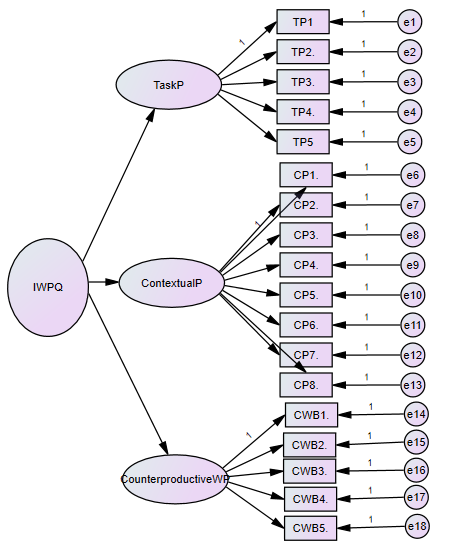


**S1 Fig. C. S1 Fig. D.**


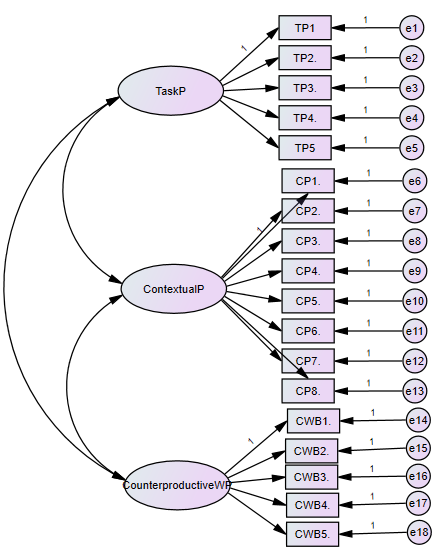

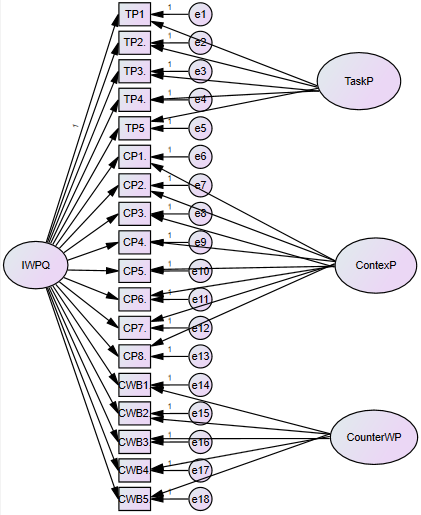

Supplement: S1 Fig — (DOCX) [file pone.0349344.s003.docx]
