# Supplementary material for: Individual work performance questionnaire: Translation and validation in Chinese
Source: PLoS One. 2026 May 15;21(5):e0349344. doi: 10.1371/journal.pone.0349344 (PMC13178909; doi:10.1371/journal.pone.0349344)

**S2 Fig.** **Standardized residual covariances.**

All standardized residuals remained below the absolute value threshold of |3.0|.


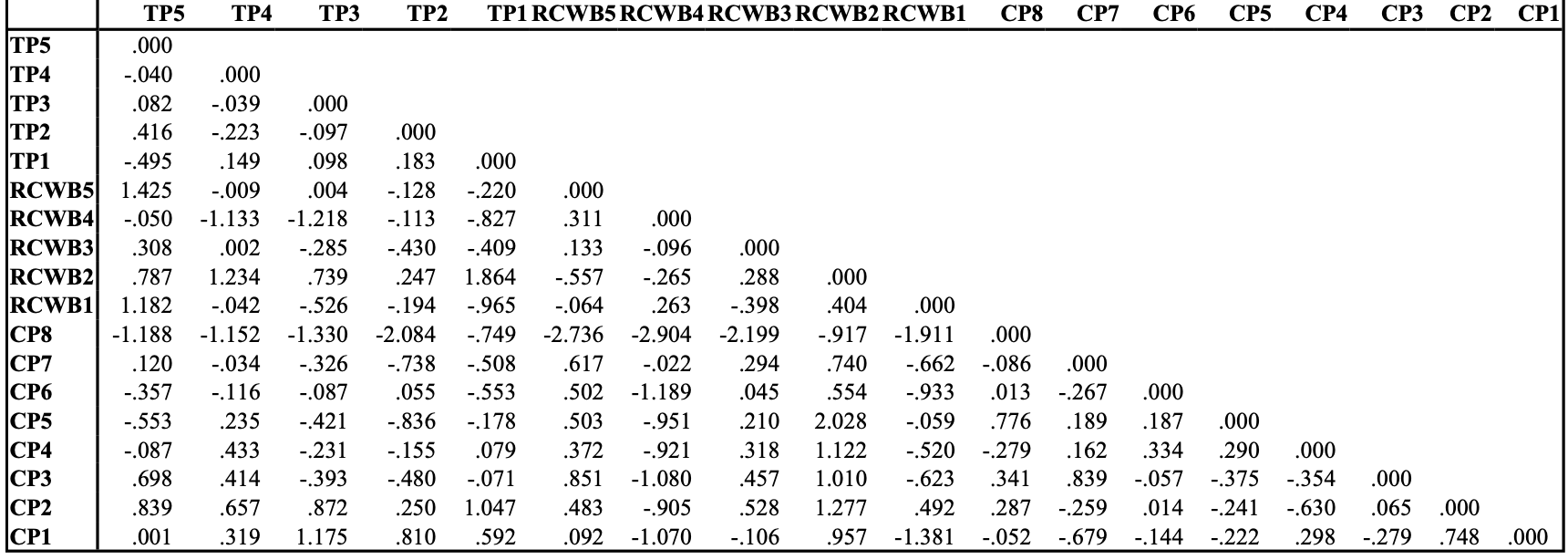

Supplement: S2 Fig — (DOCX) [file pone.0349344.s004.docx]
