# Supplementary material for: Individual work performance questionnaire: Translation and validation in Chinese
Source: PLoS One. 2026 May 15;21(5):e0349344. doi: 10.1371/journal.pone.0349344 (PMC13178909; doi:10.1371/journal.pone.0349344)

**S3 Fig. Partial Least Squares Structural Equation Model (SmartPLS).**

The model depicts the relationship between the latent variable individual work performance (IWPQ) as the independent variable and Accomplishment as the dependent variable.


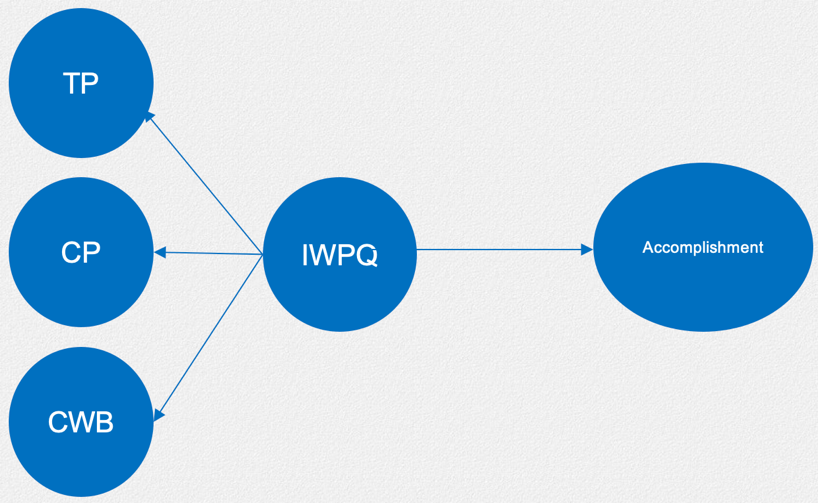

Supplement: S3 Fig — (DOCX) [file pone.0349344.s005.docx]
